# Comprehensive analysis of the role of interferon gamma-inducible protein 30 on immune infiltration and prognosis in clear cell renal cell carcinoma

**DOI:** 10.17305/bb.2023.9693

**Published:** 2024-04-01

**Authors:** Xin Wen, Lei Lei, Fan Wang, Yuan Wang

**Affiliations:** 1 Department of Pathology, Jinzhou Medical University, Jinzhou, China; 2 Department of Pathology, Affiliated Drum Tower Hospital, Medical School of Nanjing University, Nanjing, China; 3 Institute of Biological Anthropology, Jinzhou Medical University, Linghe District, Jinzhou, Liaoning, China

**Keywords:** Interferon gamma-inducible protein 30 (IFI30), clear cell renal cell carcinoma (ccRCC), immune infiltration

## Abstract

Although the immune factor interferon gamma-inducible protein 30 (IFI30) has been linked to the growth and immune infiltration of various malignancies, its function and mechanism in clear cell renal cell carcinoma (ccRCC) remains unclear. We used several databases to detect and validate IFI30 expression in ccRCC and its connection to immune invasion. We found that IFI30 expression was higher in ccRCC tissues compared to normal tissues and was strongly associated with tumor grade, T stage, and M stage. Univariate and multivariate analyses showed that ccRCC cases with lower IFI30 expression levels had a higher overall survival rate than those with high IFI30 expression (*P* < 0.05). Additionally, we collected a total of 104 cases of ccRCC and adjacent tissues from The First Affiliated Hospital of Jinzhou Medical University between January 2018 and January 2020 for immunohistochemical (IHC) analysis, along with their relevant clinicopathological data. The relationship between IFI30 and expression of *CD3E, CD4, CD8A*, interleukin 10 *(IL-10)*, and transforming growth factor beta *(TGFB2)* was examined using the ccRCC data from The Cancer Genome Atlas (TCGA) database, with findings verified by IHC analysis using the collected cases. Statistical analysis performed with SPSS found the positive correlation between the expression of C*D3E, CD4, CD8A,* and *IL-10* and the IFI30 expression, and the negative correlation of *TGFB2* expression with the IFI30 expression in ccRCC. Concurrently, a notable association was observed between high IFI30 expression and immune cell infiltration in ccRCC. High IFI30 expression is connected to the ccRCC’s poor prognosis with the infiltration of immune cell. These findings suggest that high IFI30 expression could serve as a marker of poor prognosis and be associated with immune cell infiltration in ccRCC.

## Introduction

Renal cell carcinoma (RCC) is a prevalent and histologically diverse urological malignancy [1], accounting for 3% and 5% of all cancers in men and women, respectively [2]. Clear cell renal cell carcinoma (ccRCC) is currently the most common form of RCC, followed by papillary and chromaffin cell subtypes [1]. Nowadays, with the continuous improvement of medical knowledge and treatment methods, the identification and management of RCC have significantly evolved. However, there are still many uncertainties in the development of tumors, and controversies and research issues remain [3]. Although immunotherapy is now one of the most prevalent conventional therapies for ccRCC, almost all patients eventually experience deterioration of their condition, due to the ability of ccRCC cells to resist drug-induced apoptosis [4]. Recent studies have shown that imaging may play an important role in the diagnosis and treatment of renal carcinoma, especially ccRCC. Radiogenomics has emerged as a promising agent in renal carcinoma, especially in its most common subtype ccRCC [5]. ^99m^Tc-sestamibi SPECT/CT and Girentuximab PET-CT provide faster and higher quality images to distinguish between benign and malignant lesions and different ccRCC subtypes [6]. Studies have shown that androgen receptor (AR) may influence the progression of ccRCC by affecting vascular mimicry (VM). Therefore, new anti-angiogenic therapies may also be a new direction to control the progression of ccRCC [7]. Further studies have shown that various blood test results, such as prothrombin time activity, prothrombin time, fibrinogen, etc., can better predict the prognosis of renal cancer patients of relapse-free survival [8]. Serum- and glucocorticoid-induced kinases (SGKs) promote RCC progression by mediating the phosphorylation of extracellular regulated protein kinase (ERK) 1/2 and protein kinase B (AKT/PKB). These may serve as potential prognostic markers and therapeutic targets in patients with kidney cancer [9].

Interferon gamma-inducible protein 30 (IFI30), also known as interferon gamma-inducible lysosomal thiol reductase (GILT), is an oxidoreductase associated with thiodorexin. It plays a crucial role in major histocompatibility complex (MHC) class II restriction antigen presentation and MHC class I restriction antigen cross-presentation. Additionally, IFI30 is also involved in maintaining cellular storage of the antioxidant glutathione and generating reactive oxygen species (ROS) to cope with bacterial infection [10]. The human IFI30 protein consists of 261 amino acids, including 37 amino acid signal sequences and 224 amino acid precursor forms. The 35 kDa precursor is labeled with a mannose-6-phosphate (M6P) residue and targets the endocrine pathway through the M6P receptor (M6PR) [11, 12]. IFI30 expression can induce other cell types through interferon-gamma (IFN-γ), such as melanoma cell lines [12]. Recent studies have demonstrated that a variety of immune cells frequently express IFI30, including B lymphocytes, T lymphocytes, macrophages, etc. [13, 14]. Additionally, some investigations have also explored the relationship between IFI30 and cancer, revealing that *IFI30* expression exhibits a robust relationship with immune cell infiltration and the prognosis of prostate cancer, melanoma, breast cancer, and glioma [15–18].

However, the function of IFI30 in ccRCC remains largely unexplored. In our study, we assessed the expression of IFI30 in ccRCC using various databases, including The Cancer Genome Atlas (TCGA) and the Human Protein Atlas (HPA) to obtain clinical information, and understand its relationship with the overall survival (OS) of ccRCC individuals. We utilized data from the TCGA and Tumor Immune Estimation Resource (TIMER) databases to investigate the association between *IFI30* expression and immune cell infiltration. Subsequently, we collected cases of ccRCC from The First Affiliated Hospital of Jinzhou Medical University, along with their clinical information, to corroborate our findings. Immunohistochemical (IHC) experiments revealed a significant overexpression of IFI30 in ccRCC, exhibiting a close relationship with T stage and mortality in patients with ccRCC. Moreover, our results revealed a positive correlation between *IFI30* expression and the expression of immune markers *CD3E*, *CD4*, *CD8A*, and interleukin 10 *(IL-10)* and an inverse correlation with *TGFB2* expression.

These findings suggest that high *IFI30* expression could serve as a marker of poor prognosis and be associated with immune cell infiltration in ccRCC. Our study is the first to identify IFI30 as a potential therapeutic target for immunotherapy in ccRCC.

## Materials and methods

### The Cancer Genome Atlas (TCGA)

TCGA serves as an unrestricted access to extensive cancer genomic data projects (https://genome-cancer.ucsc.edu/), providing scholars and researchers with knowledge of pathology and clinical data regarding 33 different cancers. We obtained RNA-Seq expression and matched clinicopathological information data from 269 ccRCC individuals exhibiting high *IFI30* expression levels, and 270 individuals showing low *IFI30* expression levels. We then evaluated their correlation using various clinicopathological characteristics of the individuals based on the data from TCGA browser. The database is publicly accessible and hence does not require consent by the regional ethics committee.

### The Clinical Proteome Tumor Analysis Consortium (CPTAC)

The National Cancer Institute launched the Clinical Proteome Tumor Analysis Consortium (CPTAC) in 2011 with the primary purpose of promoting the development of cancer-specific protein indicators. Its website offers scholars access on the expression of various proteins in 13 types of cancers (http://ualcan.path.uab.edu/analysis-prot.html). We utilized this resource to understand the protein expression of IFI30 in ccRCC and conducted comparative analysis.

### The Human Protein Atlas (HPA) 

Utilizing the HPA database (https://www.proteinatlas.org/), the protein levels of IFI30 in human cancers and normal tissues were analyzed.

### Tumor Immunity Estimation Resource (TIMER)

TIMER is a public website evaluating the prevalence of immune internal infiltrates in 10,897 specimens across 32 cancer types from the TCGA database (http://cistrome.org/TIMER/). Using the TIMER database, we assessed the relationship between *IFI30* expression and the abundance of six types of infiltrating immune cells (B cells, CD8+ T cells, dendritic cells, neutrophils, CD4+ T cells, and macrophages) in patients with ccRCC. Additionally, it is demonstrated how *IFI30* expression and tumor purity are correlated.

### Survival analysis

Based on the median IFI30 expression level, individuals were divided into two distinct groups: high IFI30 and low IFI30 expression groups. We created a predictive classifier for ccRCC individuals with high vs low IFI30 expression, evaluating survival differences utilizing Kaplan–Meier (KM) survival curves, to determine whether the expression levels of IFI30 influence clinical outcomes for individuals with ccRCC.

### Immuno-infiltrating T cell correlation analysis

The TCGA RNAseq data (https://portal.gdc.cancer.gov/) from the level 3 *IFI30* has been utilized using the single sample Gene Set Enrichment Analysis (ssGSEA) immune infiltration approach to determine whether its expression was connected to extensive immune infiltration of T cells.

### Inclusion and exclusion criteria

A total of 104 cases of ccRCC and adjacent tissues were collected from The First Affiliated Hospital of Jinzhou Medical University from January 2018 to January 2020. This cohort included 75 males and 29 females. IFI30 expression in ccRCC and adjacent tissues was detected by IHC.

Inclusion criteria were as follows: 1) Patients with histologically proven ccRCC; 2) Patients with ccRCC but no other malignant tumors; 3) Patients undergoing nephrectomy; and 4) Patients with comprehensive clinical and follow-up information.

Exclusion criteria were as follows: 1) Patients lost to follow-up; 2) Patients with incomplete case data; and 3) Patients who received preoperative adjuvant chemotherapy.

### Immunohistochemistry (IHC) and immunostaining used for evaluation

ccRCC specimens were dehydrated, embedded in wax blocks, and then sectioned for hydration and heat-mediated antigen retrieval using EDTA buffer at high temperature and pressure for 5 min. After endogenous peroxidase extinction and tissue blocking, ccRCC sections were cultured with antibodies against IFI30 and CD3E, CD4, CD8A, IL-10, TGFB2. This was followed by an overnight incubation at a temperature of 4 ^∘^C and 30 min with biotin-bound secondary reagents. After staining, the entire ccRCC slide was digitally scanned using a digital slice scanner at a magnification of ×400. Two experienced pathologists independently evaluated the slide, scoring the intensity and percentage of immunostaining.

### Immuno-infiltrating T cell correlation analysis

The level 3 HTSeq-FPKM format of the TCGA RNAseq data (https://portal.gdc.cancer.gov/), specifically from the kidney renal clear cell carcinoma (KIRC) project was used to analyze *IFI30*. This analysis was conducted using the ssGSEA immune infiltration approach to determine whether its expression was connected to extensive immune infiltration by T cells.

### Ethical statement

All included patients gave their oral and written informed consent. The study was approved by the Ethics Committee of JinZhou Medical University.

### Statistical analysis

The analysis and creation of the figures has been conducted using R software (version 3.6.3, https://www.r-project.org/). Patients’ characteristics have been contrasted across groups employing the Fisher’s exact test and the chi-squared test. The *P* value of OS, progression-free survival (PFS), and disease-specific survival (DSS) were determined using log-rank test for verification. For unpaired samples, Wilcoxon’s rank-sum test was utilized, and for paired samples, Wilcoxon’s signed-rank test was employed. The R survival package (https://CRAN.R-project.org/package=survival) was used to analyze IFI30 survival data and provide graphical representations of the results. Multivariate Cox analysis was used to examine the prognostic significance of the risk score, while Pearson correlation analysis was employed to identify the connection among gene expression. Time-dependent receiver operating characteristic (ROC) curve analysis was also conducted to calculate the predictive value of risk scores. The ssGSEA immune infiltration approach was employed to analyze the infiltration of *IFI30*-specific T cell, and the findings were visualized. Statistical significance was presented as *P* < 0.05.

## Results

### Reduced IFI30 mRNA and protein expression in normal tissues vs ccRCC samples

**Figure 1.**
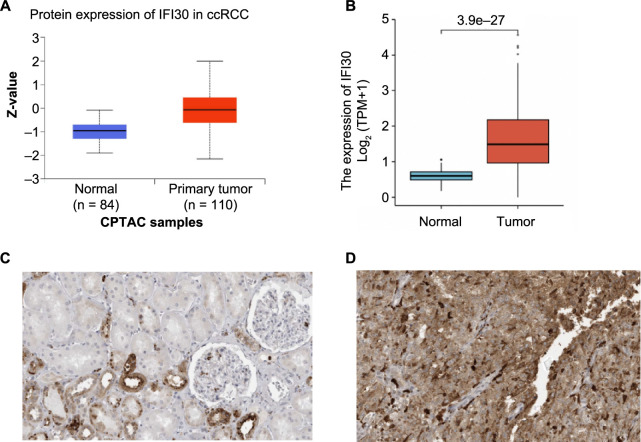
**Protein and mRNA expression of IFI30 in ccRCC samples.** (A) Across CPTAC samples, the protein expression level of IFI30 in ccRCC was significantly greater than those found in normal tissues; (B) Retrieved from the TCGA database, the RNA expression level of *IFI30* in ccRCC was significantly greater than those found in normal tissues; (C and D) In the HPA, the expression of IFI30 in ccRCC was significantly upregulated compared with normal tissues; (C) The expression of IFI30 in normal renal tissues; (D) The expression of IFI30 in ccRCC tissues. CPTAC: Clinical Proteome Tumor Analysis Consortium; TCGA: The Cancer Genome Atlas; HPA: Human Protein Atlas; IFI30: Interferon gamma-inducible protein 30; ccRCC: Clear cell renal cell carcinoma; TPM: Transcripts per million.

Utilizing the CPTAC database, the protein expression levels of IFI30 have been investigated in ccRCC. The findings demonstrated that IFI30 protein expression in normal tissues was considerably lower compared with those in ccRCC tissues (Figure 1A). In addition, in TCGA database, we normalized the fragments per kilobase of exon model per million mapped fragments (FPKM) values using the transcripts per million (TPM) method and then converted them (log2+1). We examined *IFI30* mRNA expression levels in ccRCC and found that these levels were significantly lower in normal tissues compared with ccRCC samples (*P* ═ 3.4E-24) (Figure 1B). In the HPA, IFI30 expression in human ccRCC tissues was also found considerably higher when compared with healthy tissues (Figure 1C and 1D).

### Higher *IFI30* expression mRNA showed shorter OS, PFS, and DSS in ccRCC

We divided the ccRCC data from the TCGA dataset into two distinct groups based on *IFI30* expression: high and low. We then plotted KM curves to compare their OS, PFS, and DSS.

**Figure 2.**
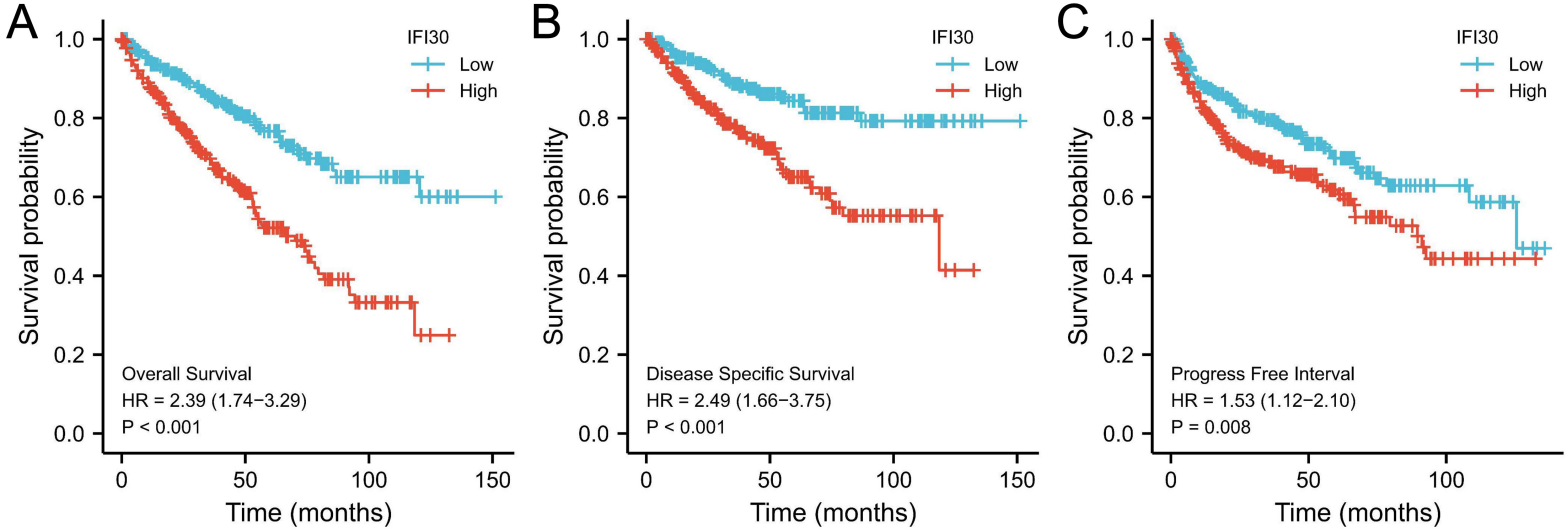
**Kaplan–Meier analysis for individuals diagnosed with ccRCC.** (A) Overall survival; (B) Disease-specific survival; (C) Progression-free survival. ccRCC: Clear cell renal cell carcinoma; IFI30: Interferon gamma-inducible protein 30.

KM plot showed that ccRCC cases with higher *IFI30* mRNA expression had shorter OS (*P* < 0.001), PFS (*P* < 0.05), and DSS (*P* < 0.001) compared with those with lower *IFI30* expression in the tested cohort (Figure 2).

### Higher *IFI30* expression levels identified as an independent predictive factor in ccRCC

**Figure 3.**
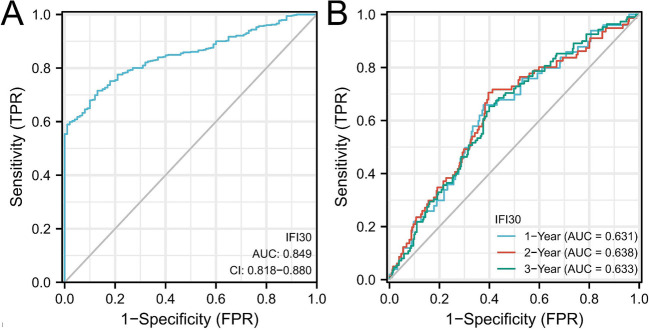
**Effect of IFI30 on ccRCC prognosis**. (A) Time-dependent ROC curve analysis has been conducted for *IFI30* expression efficiency, exhibiting an AUC of 0.849; (B) Time-dependent ROC curve analysis has been conducted for *IFI30* expression efficacy to predict 1-year, 3-year, and 5-year OS in the TCGA dataset, with AUC of 0.631, 0.633, and 0.638, respectively. AUC: Area under the curve; ROC: Receiver operating characteristic; IFI30: Interferon gamma-inducible protein 30; ccRCC: Clear cell renal cell carcinoma; TCGA: The Cancer Genome Atlas; OS: Overall survival; FPR: Fragments per million; TPR: True positive rate; CI: Confidence interval.

**Figure 4.**
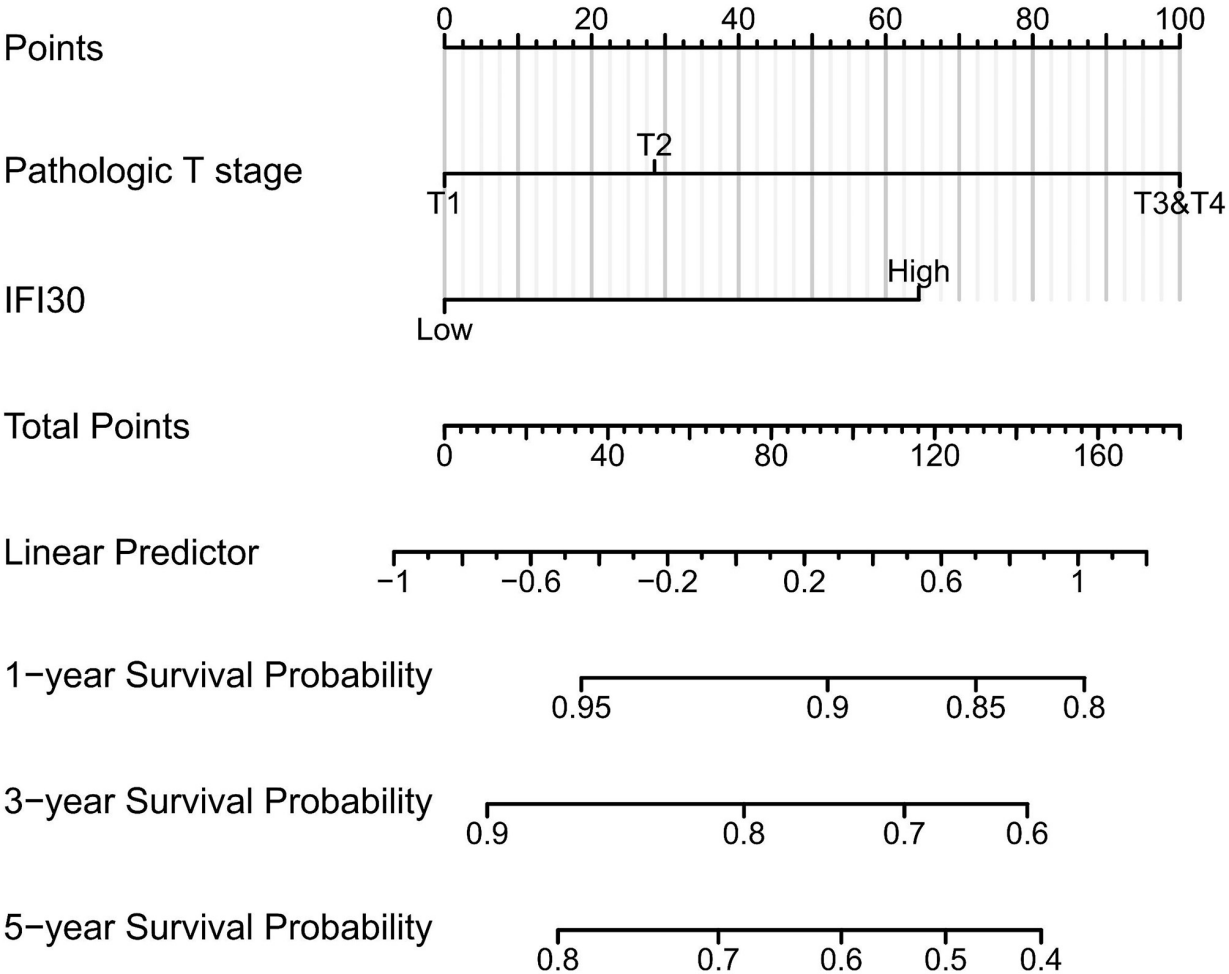
**Nomogram to predict 1-year, 3-year, and 5-year overall survival.** Each risk factor corresponds to a point by drawing a line straight upward to the points axis. The sum of the points located on the total points axis represents the probability of 1-year, 3-year, and 5-year overall survival by drawing a line straight down to the survival axis. IFI30: Interferon gamma-inducible protein 30.

**Figure 5.**
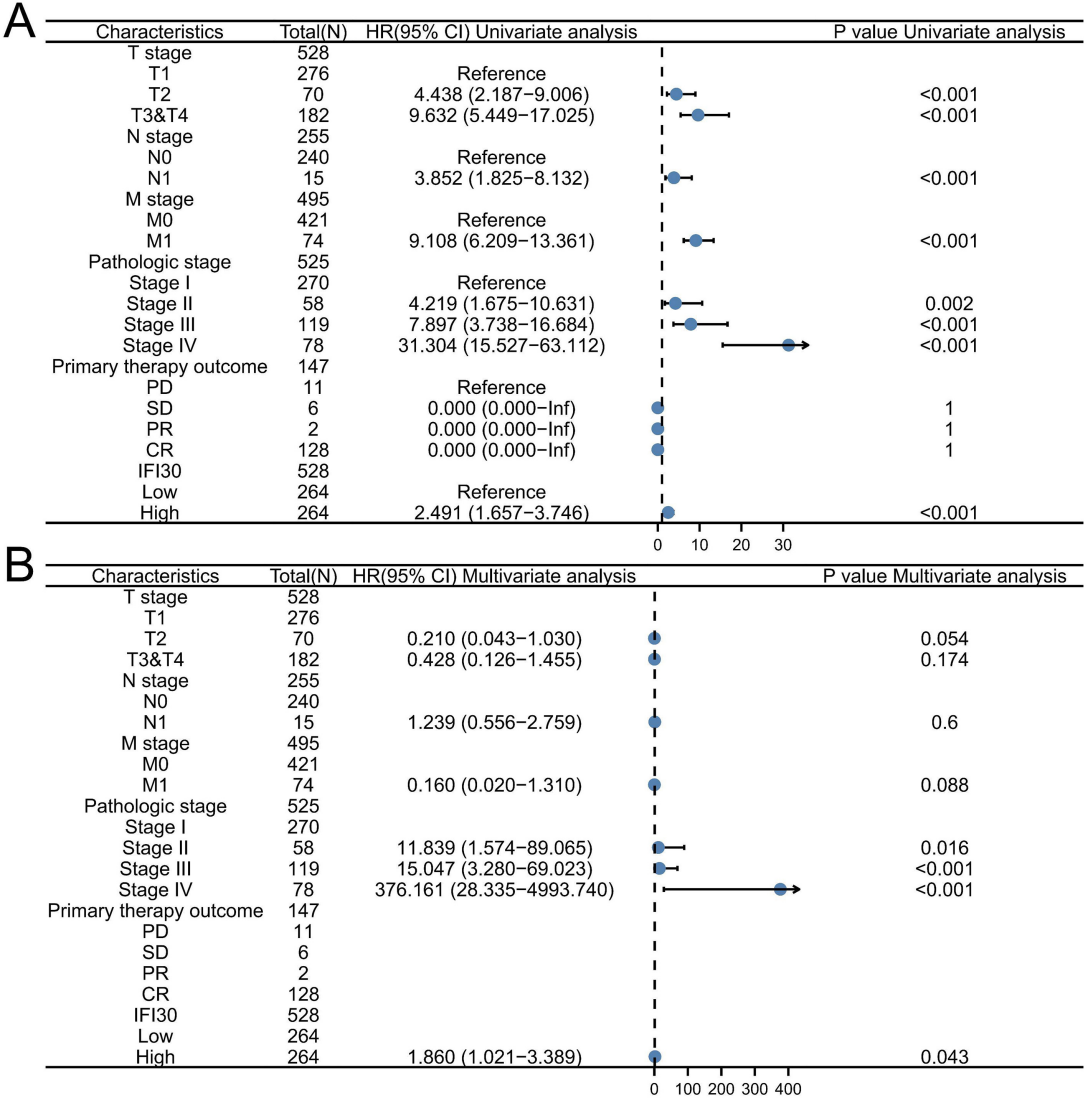
**Univariate and multivariate regression analysis of *IFI30* and other clinicopathological parameters with DSS in ccRCC individuals.** (A) The univariate Cox regression analysis results; (B) The multivariate Cox regression analysis results. IFI30: Interferon gamma-inducible protein 30; ccRCC: Clear cell renal cell carcinoma. PD: Progressive disease; SD: Stable disease; PR: Partial response; CR: Complete response.

Utilizing data from ccRCC in the TCGA database, we plotted the ROC curve (Figure 3A). The area under the ROC curve (AUC) value was 0.849, finding *IFI30* to be essential in the diagnosis of ccRCC. Using TCGA data, a time-dependent ROC curve analysis of *IFI30* expression’s predictive efficiency was performed for OS at 1, 3, and 5 years, exhibiting AUC of 0.631, 0.633, and 0.638, respectively. Based on these results, we developed prediction models and created graphical nomograms to predict the probability of 1-year, 3-year, and 5-year survival for OS (Figure 4). We then constructed univariate vs multivariate Cox models using available TCGA data, imported relevant results into forest plots, and identified that *IFI30* expression served as an independent predictive indicator for ccRCC (Figure 5). *IFI30* and other clinicopathologic characteristics were analyzed using both univariate and multivariate regression analysis alongside with DSS in patients with ccRCC (Figure 5).

### The association between *IFI30* expression and immune cells infiltration

**Figure 6.**
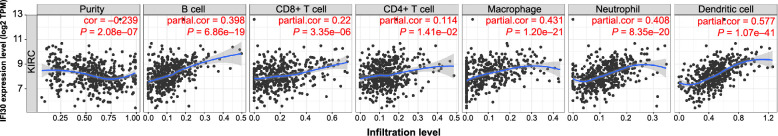
**Association between IFI30 expression and tumor immune cell infiltration (purity, B cell, CD8+ T cell, CD4+ T cell, macrophage, neutrophil and dendritic cells) in KIRC.** IFI30: Interferon gamma-inducible protein 30; TPM: Transcripts per million; KIRC: Kidney renal clear cell carcinoma.

The relationship between the expression of *IFI30* and immune cells infiltration was explored, focusing on various cell types, including CD8+ T cells, B cells, dendritic cells, neutrophils, CD4+ T cells, and macrophages. The results revealed a significant connection between *IFI30* expression levels and the levels of cell purity infiltration (*r* ═ 0.239, *P* ═ 2.8e-07), B cells (*r* ═ 0.398, *P* ═ 6.86e-19), CD8+ T cells (*r* ═ 0.22, *P* ═ 1.41e-02), CD4+ T cells (*r* ═ 0.114, *P* ═ 1.41e-02), macrophages (*r* ═ 0.431, *P* ═ 1.20e-21), neutrophils (*r* ═ 0.408, *P* ═ 8.35e-20), and dendritic cells (*r* ═ 0.557, *P* ═ 1.07e-41) in ccRCC (Figure 6), where *P* < 0.05 was reported as differentiable.

**Figure 7.**
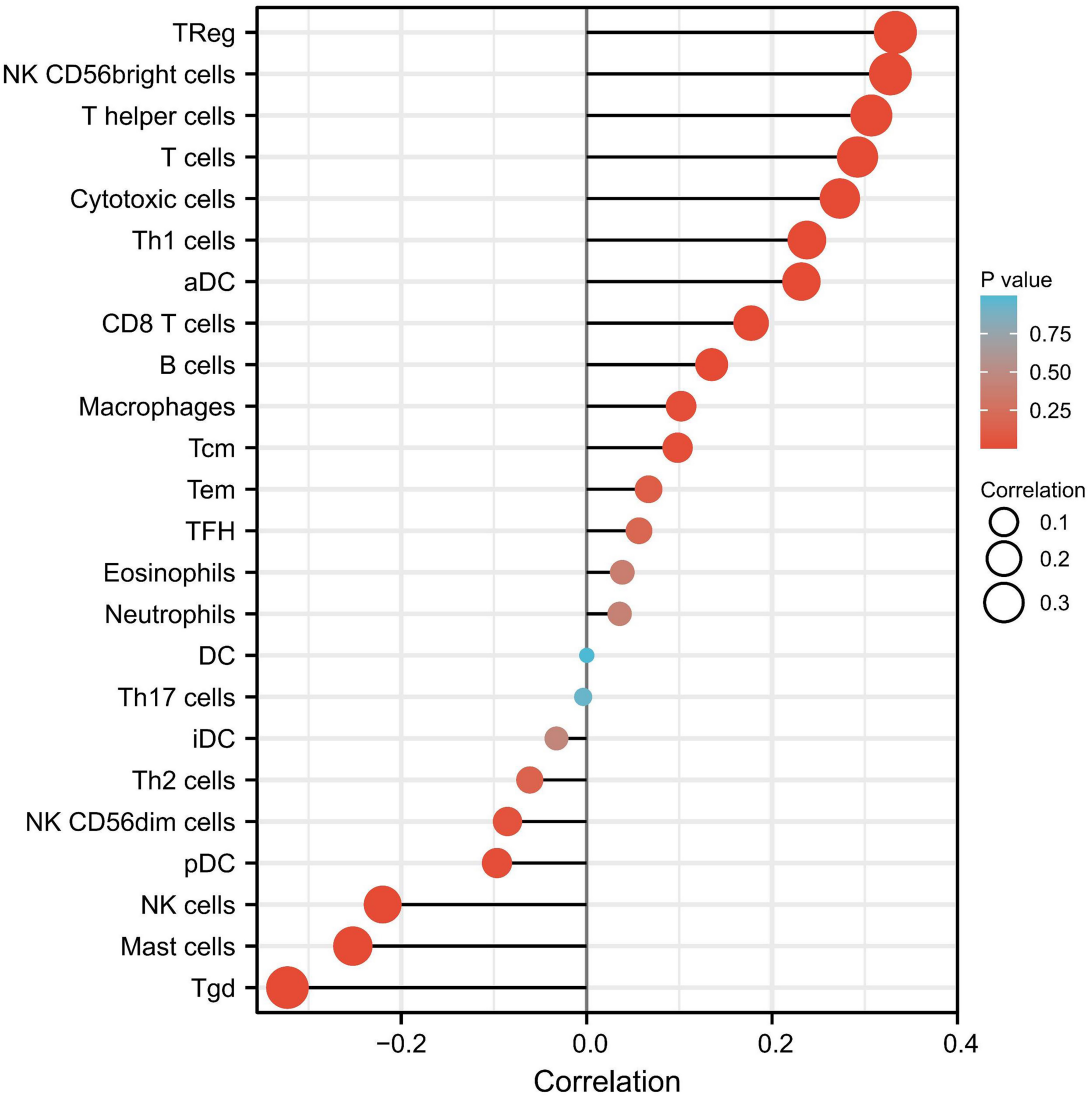
**Relationship between *IFI30* and several common immunolabeled T cells.** IFI30: Interferon gamma-inducible protein 30; aDC: Activated dendritic cell.

**Figure 8.**
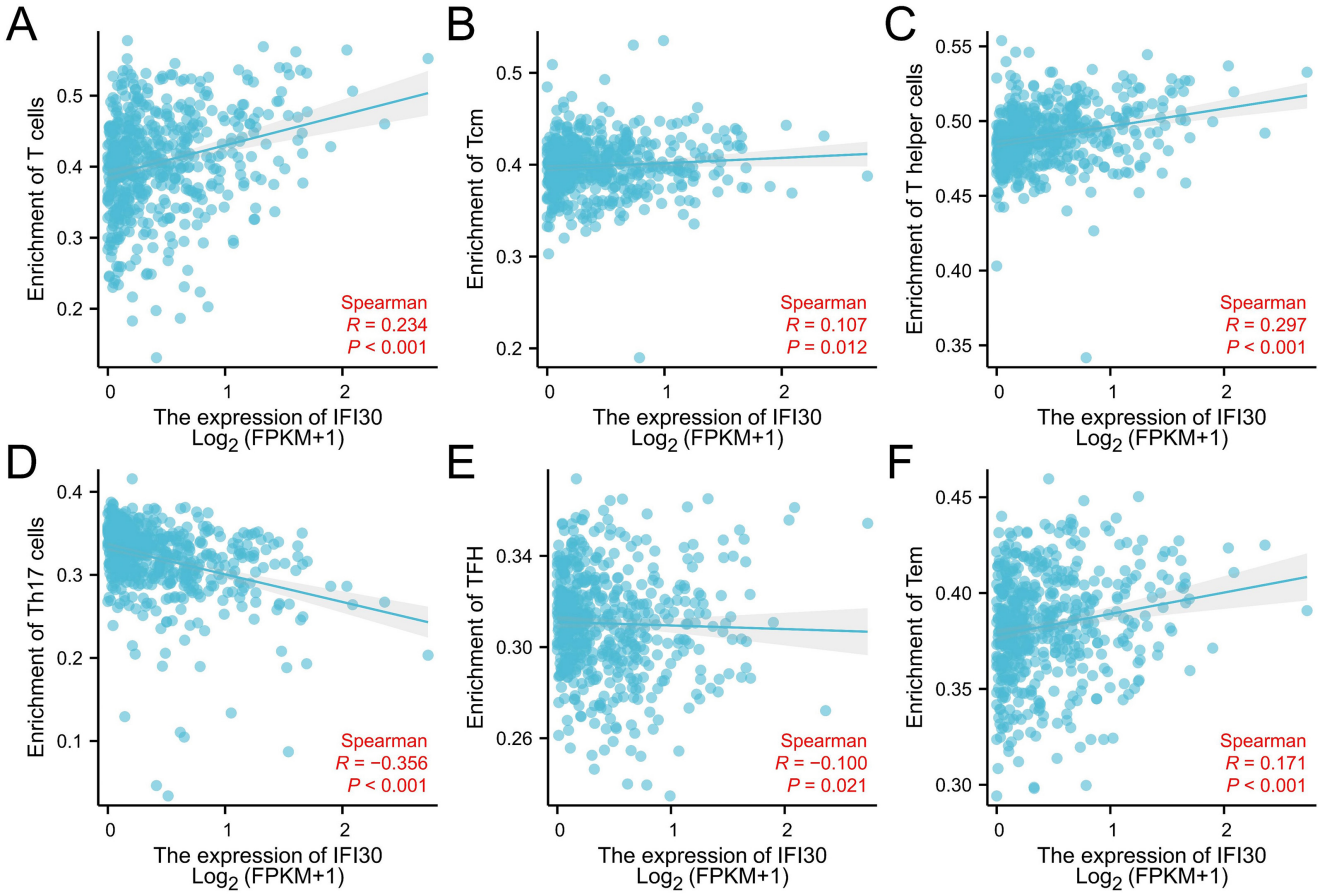
**Correlation between *IFI30* expression and various common immune infiltration-associated T cells**. (A–C) *IFI30* expression is statistically correlated to infiltration of T cells, helper T cells, and Tcm cells; (D–F) *IFI30* expression is not statistically correlated to the infiltration of Th17 cells, TFH cells, and Tem cells. IFI30: Interferon gamma-inducible protein 30; FPKM: Fragments per kilobase million.

For investigating the prospective function of *IFI30* within the penetration of different immune cells across ccRCC, we utilized ccRCC data from TCGA information to determine the connection between *IFI30* and several immunolabeled cells, such as activated dendritic cell (aDC), CD8+ T cells, cytotoxic cells, B cells, DC cells, macrophages, mast cells, eosinophils, neutrophils, NK cells, and T cells. What it showed is that aDC cells, B cells, cytotoxic cells, CD8+ T cells, mast cells, macrophages, T cells, helper T cells, gamma delta T cells cells, T helper 1 (Th1) cells, and Treg immune infiltration levels were associated to the expression of *IFI30* in ccRCC (Figure 7). These findings suggest that *IFI30* might contribute to immune infiltration within ccRCC. We also examined the connection between *IFI30* expression and various common immune infiltration-associated T cells and discovered that *IFI30* expression was statistically significantly correlated with T cells, helper T cells, central memory T (TCM) cells, T helper 17 cells (Th17), follicular helper T (Tfh) cells, and effector memory T cells (Tem) infiltration (Figure 8), with *P* < 0.05 as the cutoff for statistical significance.

### IHC analysis of IFI30 in normal and ccRCC tissues

**Figure 9.**
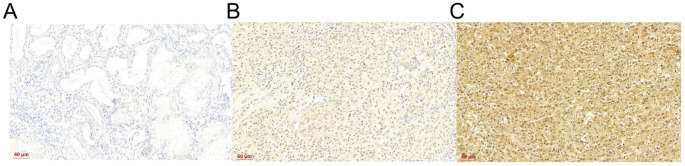
**IFI30 immunohistochemistry in normal and ccRCC tissues.** (A) IFI30 expression in normal tissues; (B) Low expression of IFI30 in ccRCC; (C) High expression of IFI30 in ccRCC. IFI30: Interferon gamma-inducible protein 30; ccRCC: Clear cell renal cell carcinoma.

A total of 104 cases of ccRCC and adjacent tissues, collected from January 2018 to January 2020 at The First Affiliated Hospital of Jinzhou Medical University, were analyzed. This cohort included 75 males and 29 females. The expression of IFI30 in RCC and adjacent tissues was detected by the IHC method. The scoring method for IHC staining was as follows: 0% ═ 0, 1%–24% ═ 1, 25%–49% ═ 2, 50%–74% ═ 3, and 75%–100% ═ 4. Overall staining was evaluated from 0 (no staining) to 1 (weak staining), 2 (moderate staining), and 3 (intense staining). The final judgment was based on the product of these two factors, with 0–6 considered as low expression and 7–12 as high expression (Figure 9). At the same time, the T stage, International Society of Urological Pathology (ISUP) grade, mortality, age, and sex of the above patients were collected and statistically analyzed (Table 1).

**Table 1:** Relationship between clinicopathological IFI30 expression and clinical data of individuals diagnosed with ccRCC

**Characteristic**	**High IFI30 expression**	**Low IFI30 expression**	***P***
n	64	40	
T stage, *n* (%)			<0.001
T1	46 (44.2%)	5 (4.8%)	
T2	10 (9.6%)	27 (26%)	
T3	5 (4.8%)	3 (2.9%)	
T4	3 (2.9%)	5 (4.8%)	
ISUP grading, *n* (%)			0.481
1	13 (12.6%)	12 (11.7%)	
2	36 (35%)	17 (16.5%)	
3	12 (11.7%)	10 (9.7%)	
4	2 (1.9%)	1 (1%)	
Survival outcome, *n* (%)			0.025
Death	24 (23.1%)	6 (5.8%)	
Survival	40 (38.5%)	34 (32.7%)	
Age (years), *n* (%)			1.000
>60	34 (32.7%)	21 (20.2%)	
≤60	30 (28.8%)	19 (18.3%)	
Sex, *n* (%)			0.457
Female	20 (19.2%)	9 (8.7%)	
Male	44 (42.3%)	31 (29.8%)	

IFI30: Interferon gamma-inducible protein 30; ccRCC: Clear cell renal cell carcinoma; ISUP: International Society of Urological Pathology.

The findings demonstrated that ccRCC exhibited IFI30 expression levels that were substantially greater than those of normal tissues. There was a notable association between IFI30 expression and T stage and mortality in ccRCC (*P* < 0.05). However, there was no significant association with ISUP grade, age, and sex (*P* > 0.05).

### Expression of IFI30 and CD3E, CD4, CD8A, IL-10, and TGFB2 in ccRCC

**Figure 10.**
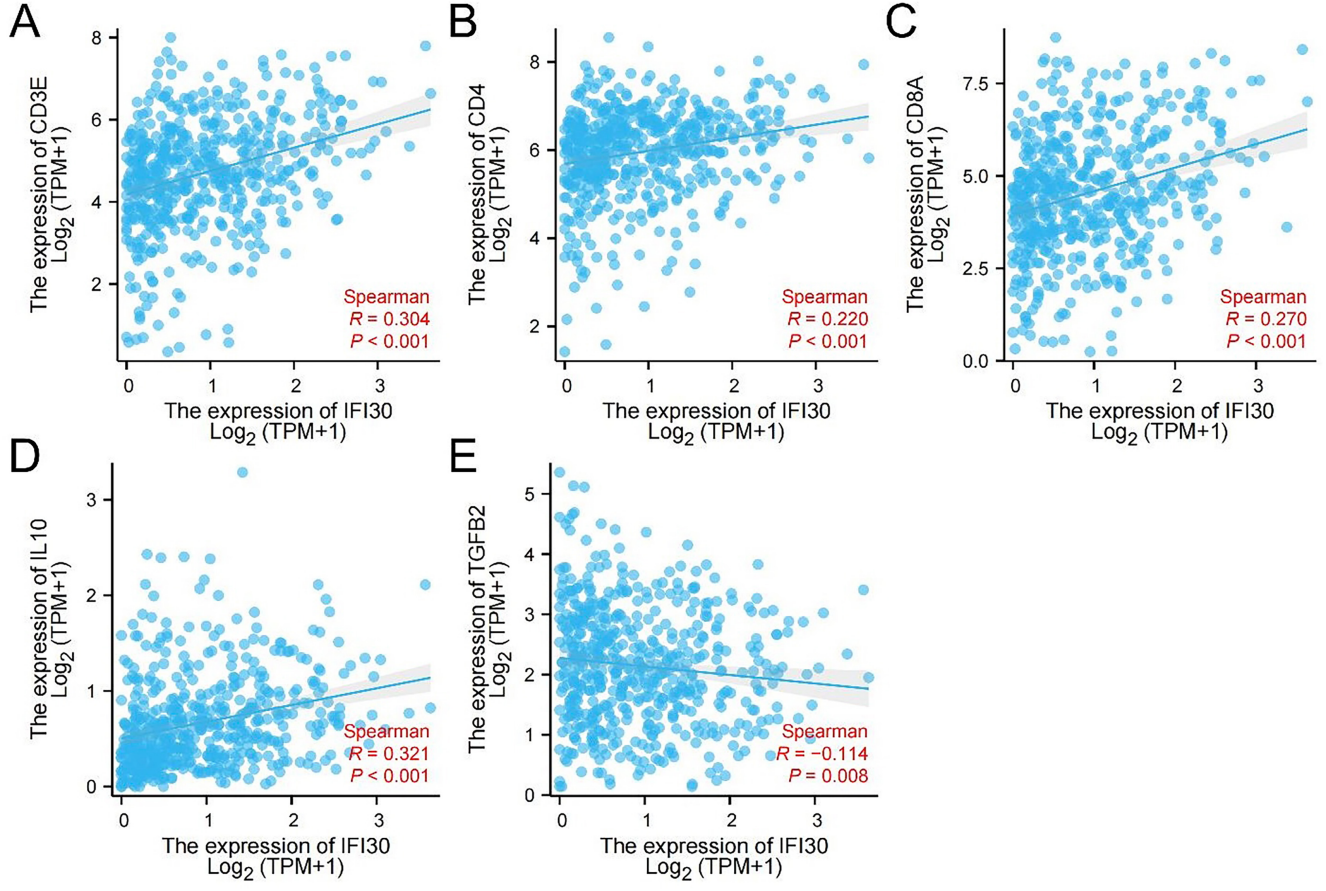
**Correlation between *IFI30* and five common immune factors in ccRCC.** (A) Correlation between *IFI30* and *CD3E*; (B) Correlation between *IFI30* and *CD4*; (C) Correlation between *IFI30* and *CD8A*; (D) Correlation between *IFI30* and *IL-10*; (E) Correlation between *IFI30* and *TGFB2*. IFI30: Interferon gamma-inducible protein 30; ccRCC: Clear cell renal cell carcinoma; IL-10: Interleukin 10; TGFB2: Transforming growth factor beta; TPM: Transcripts per million.

The ccRCC data was utilized from the TCGA database for further analysis. The investigation revealed a positive association among the expression level of *IFI30* and *CD3E, CD4, CD8A,* and *IL-10* in ccRCC. In contrast, *IFI30* expression was negatively correlated with *TGFB2* across ccRCC (Figure 10).

**Figure 11.**
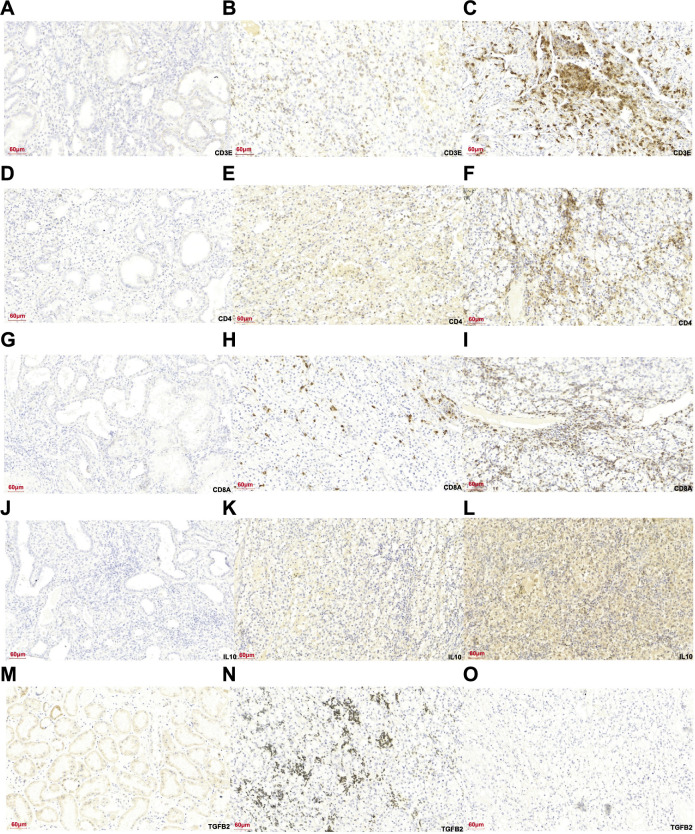
**Immunohistochemical plot of IFI30 with five common immune factors**: (A) Expression of CD3E in normal renal tissue; (B) CD3E expression ccRCC with low IFI30 expression; (C) CD3E expression in ccRCC with high IFI30 expression; (D) Expression of CD4 in normal renal tissue; (E) Expression of CD4 in ccRCC with low IFI30 expression; (F) Expression of CD4 in ccRCC with high IFI30 expression; (G) Expression of CD8A in normal renal tissue; (H) The expression of CD8A in ccRCC with low IFI30 expression; (I) Expression of CD8A in ccRCC with high IFI30 expression; (J) IL-10 expression in normal renal tissue; (K) IL-10 expression in ccRCC with low IFI30 expression; (L) IL-10 expression in ccRCC with high IFI30 expression; (M) TGFB2 expression in normal renal tissue; (N) TGFB2 expression in ccRCC with low IFI30 expression; (O) TGFB2 expression in ccRCC with high IFI30 expression. IFI30: Interferon gamma-inducible protein 30; ccRCC: Clear cell renal cell carcinoma; IL-10: Interleukin 10; TGFB2: Transforming growth factor beta.

We divided the cases into two distinct groups relied on IFI30 expression: high IFI30 expression group and low IFI30 expression. IHC staining for CD3E, CD4, CD8A, IL-10, and TGFB2 was then performed in these groups (Figure 11). Our experiments showed that the expression of CD3E, CD4, CD8A, and IL-10 in ccRCC tissues was positively linked with the IFI30 expression. On the other hand, TGFB2 expression in ccRCC tissues exhibited an inverse relationship with IFI30 expression, aligning with our expected conclusions (Table 2).

**Table 2:** Correlation between the expression of *CD3E*, CD4, *CD8A*, *IL-10*, and *TGFB2* and *IFI30* expression

IFI30	CD3E		r	*P*	CD4		r	*P*
	+	−			+	−		
+	38	26	0.542	< 0.001	42	22	0.402	< 0.001
−	9	31			12	28		
IFI30	CD8A				IL10			
	+	−			+	−		
+	43	21	0.635	< 0.001	35	29	0.648	< 0.001
−	17	23			11	29		
IFI30	TGFB2							
	+	−						
+	14	50	−0.433	< 0.001				
−	12	28						

IL-10: Interleukin 10; IFI30: Interferon gamma-inducible protein 30; TGFB2: Transforming growth factor beta.

IFI30 showed a positive correlation with the expression of CD3E, CD4, CD8A, and IL-10, while it exhibited a negative correlation with TGFB2 expression.

## Discussion

IFI30, also known as GILT, is an oxidoreductase associated with thiodoxine [9] and the only reductase identified in mammalian lysosomal vesicles [19]. When GILT was studied in the context of antigen presentation, it was found to accelerate the disulfide reduction in bonds and promote the preparation and presentation of disulfide-bonded protein antigens to antigen-specific T cells [20]. The occurrence of GILT in tumor cells has been found to enhance T cell-mediated antitumor immune surveillance and increasing the colon cancer’s GILT-MHC-I axis is an effective method of immunotherapy [21].

ccRCC is the most prevalent form of RCC. Alongside surgical intervention, innovative treatments, such as immunotherapy, are being explored [22]. Concurrently, the molecular biology of ccRCC [23] and immune infiltration have emerged as new research focal points [24]. In our study, we investigated the role of IFI30 expression in tumorigenesis and progression, as well as its impact on the prognosis of ccRCC, utilizing diverse databases such as TCGA and the HPA. By querying the CPTAC database, we discovered a significant increase in IFI30 protein expression levels across ccRCC. Furthermore, an analysis of IFI30 IHC staining in normal renal tissues and ccRCC tissues within the HPA database revealed a notable elevation in positivity rates in ccRCC tissues compared to the control group. Additionally, *IFI30* mRNA expression in ccRCC was found to be statistically associated with pathological stage, sex, T stage, histologic grade, and M stage. The KM survival curve revealed that higher *IFI30* expression was correlated with shorter OS, PFS, and DSS in ccRCC, suggesting that individuals with ccRCC and high *IFI30* expression might have an unfavorable prognosis.

We plotted ROC curves to compare the link between *IFI30* expression and RCC identification, achieving an AUC of 0.849. Additionally, we conducted time-dependent ROC curve analysis of *IFI30* expression’s efficiency for predicting OS at 1, 3, and 5 years, with AUC values of 0.631, 0.633, and 0.638, respectively. These results underscored the accuracy of *IFI30* expression in diagnosing of ccRCC. The nomogram demonstrated a strong concordance between observed results and predicted probabilities. Both univariate and multivariate Cox analyses indicated that a high *IFI30* expression is a positive predictor of DSS in patients with ccRCC. These findings imply that *IFI30* participates in the immune response and that its overexpression may accelerate the progression of ccRCC.

Based on our previous data, we observed a significant correlation between IFI30 expression and both the occurrence and prognosis of ccRCC. To validate this conclusion, we collected 104 ccRCC tissues and adjacent tissues for IFI30 IHC and obtained their related clinical data for analysis. The results showed that ccRCC had significant IFI30 expression levels. Using the varying levels of IFI30 expression as a basis, we divided the 104 individuals into high and low expression groups, and collected their T stage, ISUP grade, death cases, age, and sex for inclusion in the baseline data table. Our analysis revealed a robust association between IFI30 expression levels and T stage and mortality.

Immune infiltration in the tumor microenvironment (TME) performs a critical function within cancer development and may affect the efficacy of chemotherapy, immunotherapy, and radiotherapy, thereby affecting the prognosis of cancer patients. Relevant studies have shown a correlation between monitoring and manipulating the TME for ccRCC prognosis and precise immunotherapy [24]. In this context, our study investigated the relationship between *IFI30* expression and varying levels of immune cell infiltration in the TME. Moreover, our study revealed that the expression level of *IFI30* was closely connected to the infiltration of B cells, CD8+ T cells, dendritic cells, neutrophils, CD4+ T cells, and macrophages. Further analysis of infiltrating lymphocytes revealed that *IFI30* expression level has been significantly connected to the infiltration of activated dendritic cells (aDC), B cells, CD8+ T cells, cytotoxic cells, macrophages, and mast cells. The high permeability rate of CD8+ in TME [25] was directly related to poor prognosis in ccRCC [26]. This finding connects *IFI30* expression to the immune infiltration and prognosis of ccRCC. Additionally, our investigation into immune-infiltrating T cells revealed a strong correlation between *IFI30* expression level and the infiltration of T cells, helper T cells, and Tcm cells, indicating that *IFI30* may have a regulating function in tumor-associated macrophage (TAM) polarization. The association among the immune microenvironment of ccRCC, T cells infiltration and the therapeutic impact of tumors have recently been examined using single-cell sequencing, demonstrating that the T cells infiltration has significant implications to the prognosis of ccRCC [19].

To further understand about the connection between *IFI30* and TME, it has been observed that chronic inflammation and immune response are two fundamental components of the TME. There is an extensive quantity of immune/inflammatory cells (involving TAMs, neutrophils, and myeloid-derived suppressor cells) and cytokines (involving TGF-β, IL-6, and IL-10) in the TME, which lead to a chronic inflammatory state and immune suppression [27]. Studies have shown that *IFI30* plays a substantial part in shaping the tissue-restricted autoantigens of CD4+ T cells in mediating autoimmune diseases and anti-tumor immunity [28]. Several investigations have also demonstrated that *IFI30* has the ability to enhance the expression of MHC class class I which is vital in antigen cross-presentation. This enhancement inhibits the identification of these antigens by CD8+ T cells and is closely related to the process of antigen presentation [29]. IL-10 is a multifunctional immunomodulatory cytokine secreted from immune cells including T lymphocytes and natural killer cells, macrophages, which can also induce tumor cell progression and metastasis via immunosuppression [30]. Analysis showed that *IL-10* was a gene with predictive significance within ccRCC microenvironment [31]. According to the studies of TGF-β pathway, TGF-β can inhibit and weaken the invasion ability of ccRCC cells [32]. Therefore, *CD3E*, *CD4*, and *CD8A* were selected to label relevant immune cells, while *IL-10* and *TGFB2* were used to label cytokines for subsequent studies.

We first used the ccRCC data in the TCGA database for analysis. According to our research, *IFI30* and the expression showed a positive correlation with *CD3E*, CD4, *CD8A*, and *IL-10* in ccRCC, while *IFI30* was negatively correlated with *TGFB2* in ccRCC. Subsequently, the cases were divided into high and low *IFI30* expression groups. This division was applied to 104 collected cases of ccRCC. IHC staining of CD3E, CD4, CD8A, IL-10, and TGFB2 was performed in these two groups, and the results were analyzed in relation to IFI30 expression levels. Our experiments revealed that the expression of CD3E, CD4, CD8A, and IL-10 in ccRCC tissues exhibited a positive correlation with IFI30 expression. In contrast, TGFB2 expression in ccRCC tissues was negatively correlated with IFI30 expression, which was consistent with our expected conclusion.

## Conclusion

Our research and analysis initially established a close association between elevated *IFI30* expression and the incidence, progression, and immune infiltration of ccRCC. *IFI30* could subsequently serve as a targeting factor for ccRCC treatment, warranting further research and exploration. However, significant limitations of our study include the lack of large sample size of tumor and normal samples from databases, platform differences, and potential errors in data collection. Furthermore, the demographic scope of the TCGA database, which is primarily focused on white and black populations, poses a challenge in extending these findings to other ethnic groups. Additional literature searches are required to determine the association between *IFI30* expression and other genes. Regarding the specific role of *IFI30* on cancer prognosis and its influence on immune infiltration, although we investigated the effect of *IFI30* on immune infiltration through several key cytokines, additional experiments are necessary to verify whether IFI30 could be an effective target and predictor in cancer immunotherapy.

## Supplemental data

Supplementary data are available at the following link:

https://docs.google.com/spreadsheets/d/1LuVqVLbHhVr726_kiwlw2xE_3_JVUSyP/edit?usp=sharing&ouid=111634499466144347170&rtpof=true&sd=true

## Data Availability

All data generated or analyzed during this study are included in this article.
